# Supplementary material for: A Comparative Study of Approaches to Improve the Sensitivity of Lateral Flow Immunoassay of the Antibiotic Lincomycin
Source: Biosensors (Basel). 2020 Dec 3;10(12):198. doi: 10.3390/bios10120198 (PMC7761767; doi:10.3390/bios10120198)
Supplement: Supplementary file 1 [file biosensors-10-00198-s001.pdf]

biosensors

MDPI

Supplementary Materials: A Comparative Study of Approaches to Improve the Sensitivity of Lateral Flow Immunoassay of the Antibiotic Lincomycin

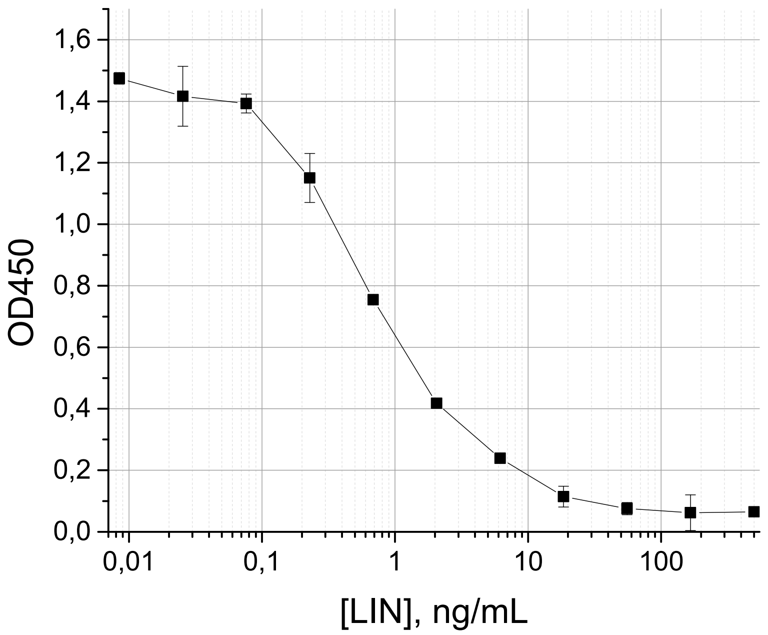

Figure S1. Competitive curve for the ELISA of LIN. The detection limit of LIN (IC10) is 0.08 ng/mL, its concentration causing 50% inhibition of the antibody binding (IC50) is 0.69 ng/mL. The error bars indicate the standard deviations for three measurements.

© 2020 by the authors. Submitted for possible open access publication under the terms and conditions of the Creative Commons Attribution (CC BY) license (http://creativecommons.org/licenses/by/4.0/).
